# Correction of poliomyelitis foot deformities with Ilizarov method

**DOI:** 10.1007/s11751-011-0111-6

**Published:** 2011-08-02

**Authors:** Alexander Kirienko, Andrea Peccati, Ibrahim Abdellatif, Yasser Elbatrawy, Zayed Mahmoud A. Mostaf, Valentina Necci

**Affiliations:** 1 Università degli Studi di Milano, Milan, Italy; 2 Istituto Clinico Humanitas, Rozzano, Italy; 3 Alzahraa University Hospital, Cairo, Egypt; 4 Università degli Studi di Roma “La Sapienza”, Rome, Italy; 5 Azienda Ospedaliera S. Andrea, Rome, Italy; 6 Studio Medici Ovest 2, Istituto Clinico Humanitas, Via A. Manzoni, 56, 20089 Rozzano (Milan), Italy

**Keywords:** External fixation, Foot deformities, Ilizarov, Poliomyelitis, Surgical techniques

## Abstract

Poliomyelitis is an infectious disease caused by a neurotrophic virus targeting anterior horn cells of lower motor neurons resulting in flaccid paralysis and represents a common condition in developing countries, and even nowadays, most of both treated and untreated cases result in foot deformities. Between 1994 and 2007, 27 patients were treated by classic ring Ilizarov fixator, aiming at producing a stable plantigrade and cosmetically acceptable foot and followed up for meanly 7.17 years. Additional procedures were performed if needed. The mean time in frame was 4.2 months. All the patients were satisfied with their gait, compared to preoperative status. A painless and plantigrade foot was obtained in all patients, and limb-length discrepancy was always corrected where present. No major complications were encountered. In conclusion, the Ilizarov method allows simultaneous progressive correction of all components of severe foot deformities associated with limb-lengthening discrepancy with minimal surgery, reducing risks of cutaneous or neurovascular complications and avoiding important shortening of the foot.

## Introduction

Poliomyelitis is an infection caused by a neurotrophic virus that targets the anterior horn cells of lower motor neurons resulting in a flaccid paralysis. Evidence in the withered and deformed limbs of some mummies from ancient Egypt suggests the condition to be present over 3,000 years ago (Fig. 1) [1]. According to data available on the WHO website, poliomyelitis has been virtually eradicated as a result of worldwide immunization programmes, remaining endemic in four countries only (Afghanistan, India, Nigeria and Pakistan) and a further four countries known to have or suspected of having a re-established transmission of the poliovirus; despite this, an estimated 650,000 middle-aged patients in the United States still suffer the long-term sequelae of this disease, including lower-limb deformities and degenerative joint disease [2].

**Fig. 1:**
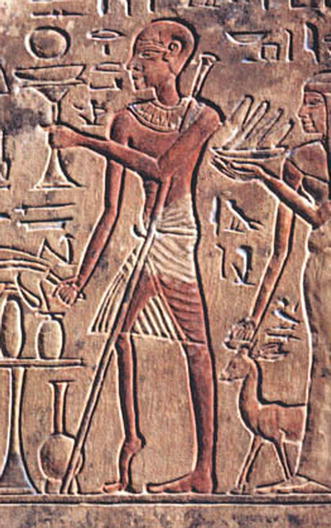
An Egyptian stele thought to represent a polio victim, 18th Dinasty (1403–1365 BC)

The long-term consequence of paralysis from poliomyelitis is deformity, the location and type depending on the muscles affected and imbalance between muscle groups. With skeletal growth, leg shortening, soft tissue contractures and foot deformities often develop in poliomyelitis [3–5]. Treatment strategies require consideration of several factors, including the instability from muscle imbalance, the presence of knee and hip contractures or poor soft tissue conditions from previous surgery. The goal of orthopaedic surgical treatment is to obtain a painless plantigrade and stable lower limb. Conventional operative techniques include corrective osteotomies, arthrodeses, extensive release of contractures and tendon transfers [5–8]. The extent of acute correction in severe deformities may result in neurovascular complications and wound healing problems. Added to this, some lower-limb problems require simultaneous leg lengthening and foot deformity corrections together with tendon transfers to augment control of the stabilized foot [9–11].

The Ilizarov method, based on the principles of distraction osteogenesis, is a treatment option for poliomyelitic foot deformities. It is considered safer than conventional methods of the treatment and may offer more predictably satisfactory results [12–15].

We report on our experience with post-poliomyelitis foot deformities and illustrate the surgical techniques and approach we use. We believe that this detailed description of the surgical techniques, based on the Ilizarov method, for the treatment for different foot deformities in polio patients has not been published previously.

## Patients and methods

Between 1994 and 2007, 27 patients with 27 deformed rigid feet were treated in San Raffaele Hospital (Milano), Istituto Clinico Humanitas (Milano) and Alzahraa University Hospital (Cairo) using the Ilizarov method and the classic Ilizarov ring fixator. The mean age was 38.96 years (range 17–66). Table 1 lists the details of the patients, including previous surgeries and type of initial deformity. Ten of the 27 patients had undergone previous surgical intervention: stabilizing procedures of the foot (5 triple arthrodesis, 1 ankle arthrodesis, 1 calcaneal-cuboid arthrodesis); four patients had undergone corrective osteotomy of femur and 2 patients corrective osteotomy of proximal tibia; 1 patient had a knee arthrodesis; 6 patients had a soft tissue release performed.

**Table 1:** Patients details

#	Age	Gender	Side	Previous surgical treatment	Initial deformity	Limb len. discrepancy (cm)
1	17	F	R	Tendon transfer	C, E, S, V, walking on dorsolateral	None
2	18	M	R	None	E, V, walking on toes	None
3	25	F	R	None	E, V	2
4	27	F	L	None	C, E, S, V, walking on dorsolateral	3
5	31	F	L	Tendon transfer	E, instable ankle, dislocation, V	None
6	23	F	R	None	E	3
7	26	M	R	Tendon transfer	E	2.5
8	17	M	L	None	C, E, V	None
9	46	F	L	Chopart arth. Subtalar arth.	Flatfoot, flexed genu valgu	2
10	36	F	R	None	Instable ankle joint, talus, V	4
11	41	M	L	Triple arth.	V ankle joint	3
12	44	M	R	None	Recurvatum knee, V, E	3
13	37	F	R	None	Genu valgum, V, S, instable foot	1.5
14	46	F	R	None	C, S, V, subtalar OA	3
15	53	F	R	Subtalar arth.	C, genu valgum, talus, V	2
16	17	F	R	None	Flatfoot	4
17	43	F	R	Triple arth.	Dislocation of ankle joint, V	3
18	48	M	R	Triple arth.	E	4
19	36	F	R	Triple arth.	E, V	3
20	37	F	R	Triple arth.	Position in dorsal flexion, TC	2
21	50	F	L	Proximal femur ost., arthroscopy	E, knee OA	4.5
22	40	M	R	None	E, flexed knee	6
23	55	F	R	None	Tibiotarsal joint OA, E, subtalar joint OA, V	3
24	66	F	R	Triple arth.	Ankle joint OA, C, E, painful tibiotalar subankylosis, V	2.5
25	59	M	R	None	C, E, V	6
26	54	F	R	Tendon transfer	Flexed genu valgum, instable foot, tibia valga, V	2
27	60	M	L	Femur ost.	Flexed knee, V	4

*arth* arthrodesis, *C* cavus, *E* equinus, *OA* Osteoarthritis, *P* Pronation, *S* Supination, *TC* Talipes calcaneus, *V* varus

The type of deformities encountered before surgery was 6 equinus feet; 4 equinovarus; 5 equinovalgus; 2 supinated equinovarus; 3 equinocavovarus; and 2 calcaneovalgus. In 15 patients, a limb-length discrepancy was observed ranging from 2 to 6 cm (mean 3.5 cm). A preoperative evaluation of both lower extremities consists of ROM measurement, muscles power evaluation, clinical photography, orthogonal plane X-rays of the foot and ankle, the knee in maximum extension, and long film views to assess deformity and limb-length discrepancy. The results were reported using descriptive statistics only as is appropriate for a review of this nature.

## Operative technique

Treatment for post-poliomyelitic deformities must be individualized owing to the diversity in type and severity. The treatment options for the foot and lower limb are the following:

1. correction of foot deformities by closed methods;
2. stabilization of the foot by arthrodesis;
3. open treatment (osteotomies) for foot deformities;
4. arthroereisis;
5. leg lengthening and deformity correction.

### Closed method

The closed method for correction of foot deformities is indicated when the equinus deformity is acquired consequent to an adaptation for a leg-length discrepancy. These patients usually walk with the aid of orthotics or modified shoes, maintaining the equinus position to compensate for limb shortening.

This method should be performed together with the leg lengthening:

- If an Achilles tendon lengthening (through gradual elongation in the fixator) is performed to correct the equinus while tibia and fibular lengths remain unaltered, the patient will experience a recurrent equinus deformity to compensate for the persistent limb-length discrepancy.
- If limb lengthening is performed alone, the patient may be subject to a worsening of the equinus deformity as lengthening proceeds.

In such cases, overcorrection of the deformity is important in anticipation of ‘spring-back’ after fixator removal. With equinus deformities, the overcorrection is by 10°–15°, whereas in multidirectional deformities, the aim is to overcorrect the varus component by 20° valgus, the cavus by 10° planus, adduction of the forefoot by 30°–35° of abduction and plantar flexion into 25°–30° dorsiflexion and supination by 20° pronation.

#### Operative technique

The fixator construct is made up of two sections: one applied to the tibia and a foot section affixed to the hindfoot, midfoot and forefoot parts. These sections are connected by hinges and rods and are set to mirror the equinus deformity. Two hinges (one medial and one lateral) are positioned exactly on the of flexion–extension axis (Inman’s axis) of the ankle. The direction of traction forces will need to be adjusted as correction progresses and the status of the ankle joint checked by lateral X-rays periodically in case joint subluxation occurs.

Occasionally and in severe contractures, the application of the apparatus may be preceded by a percutaneous lengthening of the Achilles tendon. After tenotomy, the foot is forced into dorsiflexion as far as will be allowed through the gliding sections of the tendon. The remaining equinus is then dealt with by correction through the fixator. Distraction is started after wound healing, which usually takes 1–2 weeks.

### Stabilization of the foot by arthrodesis

This technique is performed for those patients who have never been treated or on those who have undergone previous attempts at correction with suboptimal outcomes.

#### Arthrodesis of the tibio-talar joint

##### Operative technique

The joint is approached laterally. The fixator is constructed with one or two rings in the tibia. A fibular osteotomy is performed proximal to the ankle joint and turned to allow a lateral arthrotomy of the ankle for cartilage and subchondral bone removal. The foot is placed plantigrade or in a slight equinus position and temporarily fixed with two Kirschner wires introduced from the calcaneus into the tibia. The fibula is reduced and compressed against the tibia and talus with a lateral-to-medial olive wire. It is inserted at a slight oblique angle from inferior to superior, from lateral to medial and from posterior to anterior. It is connected to the tibial ring assembly via grooved (slotted) threaded rod. Two crossed wires are inserted into the talus, tensioned and tightly attached to a horseshoe-shaped (5/8) ring that is centred on the foot and is parallel to the sole of the foot. The horseshoe-shaped ring is further stabilized with two calcaneal olive wires, two midfoot wires and two metatarsal opposing olive wires connected to an anterior extension to the 5/8 ring using straight plates. The straight plates are prevented from converging when the wires are tensioned by a link across the front of the foot using threaded rods on posts. (Fig. 2) Three rods connect the horseshoe-shaped ring and tibial assembly; an additional anterior rod is used between the transverse link across the straight plates and tibial assembly to complete a circumferential connection between tibia and foot. All the rods terminate at the horseshoe-shaped ring with mobile joints in the sagittal plane in order to control the amount of equinus. Consolidation usually occurs within 45–60 days. Radiographs usually show a loss of the tibio-talar contact line and moderate callus formation as consolidation occurs in situ. Once the apparatus is removed, non-weight bearing without foot flexion is advocated for 1 month in order to avoid placing the arthrodesis under undue stress. In the case of uncooperative or obese patients, a weight-bearing plaster cast can be used for approximately 30–40 days.

**Fig. 2:**
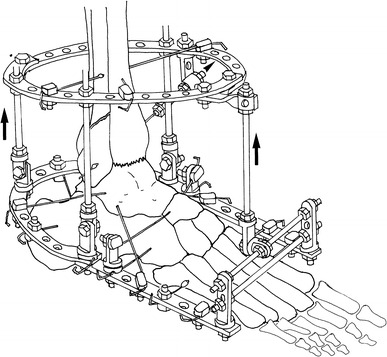
Ilizarov frame for ankle arthrodesis

#### Subtalar arthrodesis

This technique is indicated for varus or valgus paralytic calcaneus.

##### Operative technique

The tibial fixator assembly is built as previously described.

Lateral exposure of the subtalar joint and the sinus tarsi is performed for cartilage and subchondral bone resection from the posterior, anterior and middle articular facets of the talus and calcaneum. Two crossed wires are placed into the talar body. The talar wires are placed 8–10 mm above the cranial surface of the horseshoe-shaped ring. The calcaneal wires are placed 8–10 mm under the caudal surface of the ring. The wires are then clamped under tension directly (thereby arching the wires) to the horseshoe-shaped ring, which, in turn, compresses the subtalar joint. The tension of the wires causes the arch to flatten as tensioning occurs (Fig. 3). If the subtalar joint is subluxed prior to surgery, appropriate removal of osteochondral surfaces will create a space that allows acute correction. Early weight bearing is allowed, and solid consolidation will occur in about 45–60 days.

**Fig. 3:**
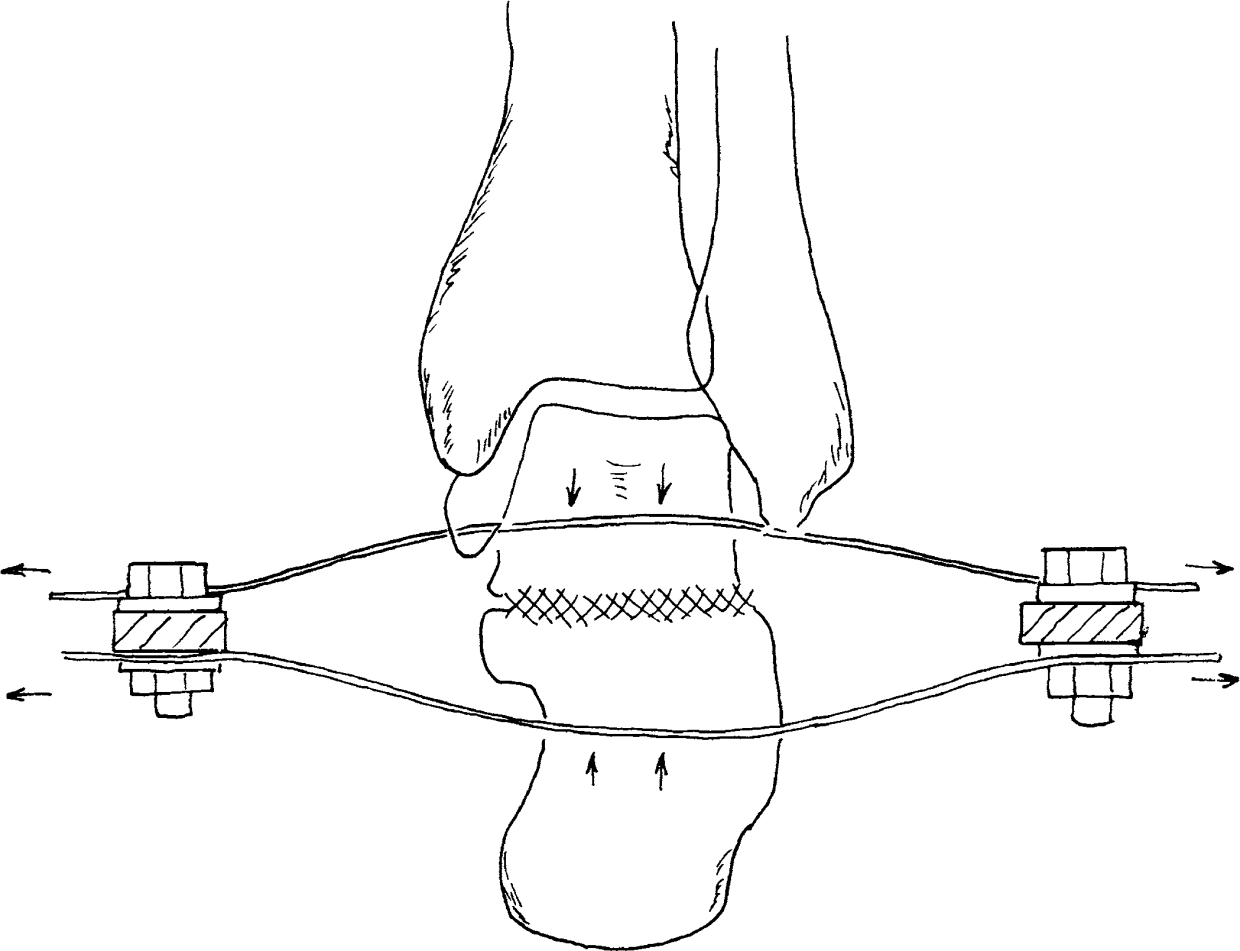
Compression on subtalar joint by arching and tensioning the wires

#### Triple arthrodesis

##### Operative technique

In this technique, surgical access is from the lateral side in the submalleolar area and extends in an anterior direction (Ollier-type approach). Cartilage is removed from the three articulations in the following order: calcaneal-cuboid articulation, talo-navicular articulation and the subtalar joint.

Two crossed wires are introduced into the talus, and three opposing olive wires are introduced into the calcaneus. The skin should be pulled away from the subtalar joint and held tight during wire introduction. A wire is inserted into the cuboid and another into the navicular, running parallel to the sole of the foot; the skin should be pulled towards the forefoot. Two opposing olive wires are introduced into the metatarsals. A horseshoe-shaped ring is placed between the wires in such a way that the talar and navicular wires are on the cranial side and the calcaneal and cuboid wires are on the plantar side, while the metatarsal wires are randomly fixed onto the straight plates. Compression is exerted on the subtalar joint by means of arching the talar wires down to the plantar ring and arching the calcaneal wires up to the plantar one. Similarly, compression is exerted on the talar-navicular articulation and on the calcaneal-cuboid articulation by fixing and tightening the wires inserted in the navicular and the cuboid into more posterior holes of the horseshoe-shaped ring, thereby achieving compression through arching and subsequent tensioning (Fig. 4).

**Fig. 4:**
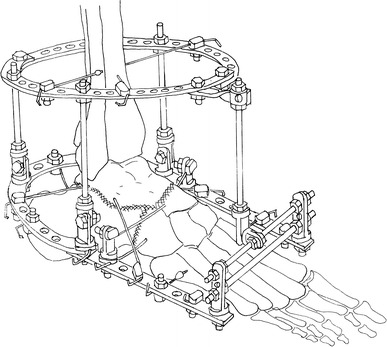
Ilizarov frame for triple arthrodesis

The technique of subtalar and triple arthrodesis with the Ilizarov fixator, concomitant with limb lengthening, assures greater stability during distraction and prevents secondary deformity of the foot. A second advantage is that no hardware remains in the foot after removal of the Ilizarov device.

### Open treatment with osteotomies

This technique in indicated as a first-choice treatment option for severe cases or those having had surgery but with significant residual deformities present. This technique can involve a *V*- or a *Y-*shaped osteotomy.

In open treatment using osteotomies, large degrees of overcorrection are not necessary, and only small amounts are needed, as there is minimal relapse of the previous deformity after removing the frame.

#### *V-*shaped osteotomy

The *V-*shaped osteotomy is indicated whenever lengthening and deformity corrections are required simultaneously. This shape of osteotomy is the result of a combination of an oblique osteotomy of the posterior calcaneus and the anterior calcaneal-talar neck osteotomy. It is indicated for the simultaneous correction of deformities posterior to the Chopart joint.

##### Operative technique

The tibial assembly is as described previously. The two osteotomy cuts intersect with an acute angle of about 60–70° on the planter surface of the calcaneus. Two opposing olive wires are placed into the talar body and are attached to the tibial assembly. The calcaneal half-ring is stabilized by four to five opposing olive wires. The metatarsal half-ring is perpendicular to the longitudinal axis of the forefoot. The distal fragment of the calcaneal osteotomy is anchored with an olive wire. A plantar fascia release is not always needed with the *V-*shaped osteotomy unless there is a significant cavus deformity but transphalangeal pinning of the toes is necessary to prevent the development of claw-toe deformities (Figs. 5, 6, 7, 8).

**Fig. 5:**
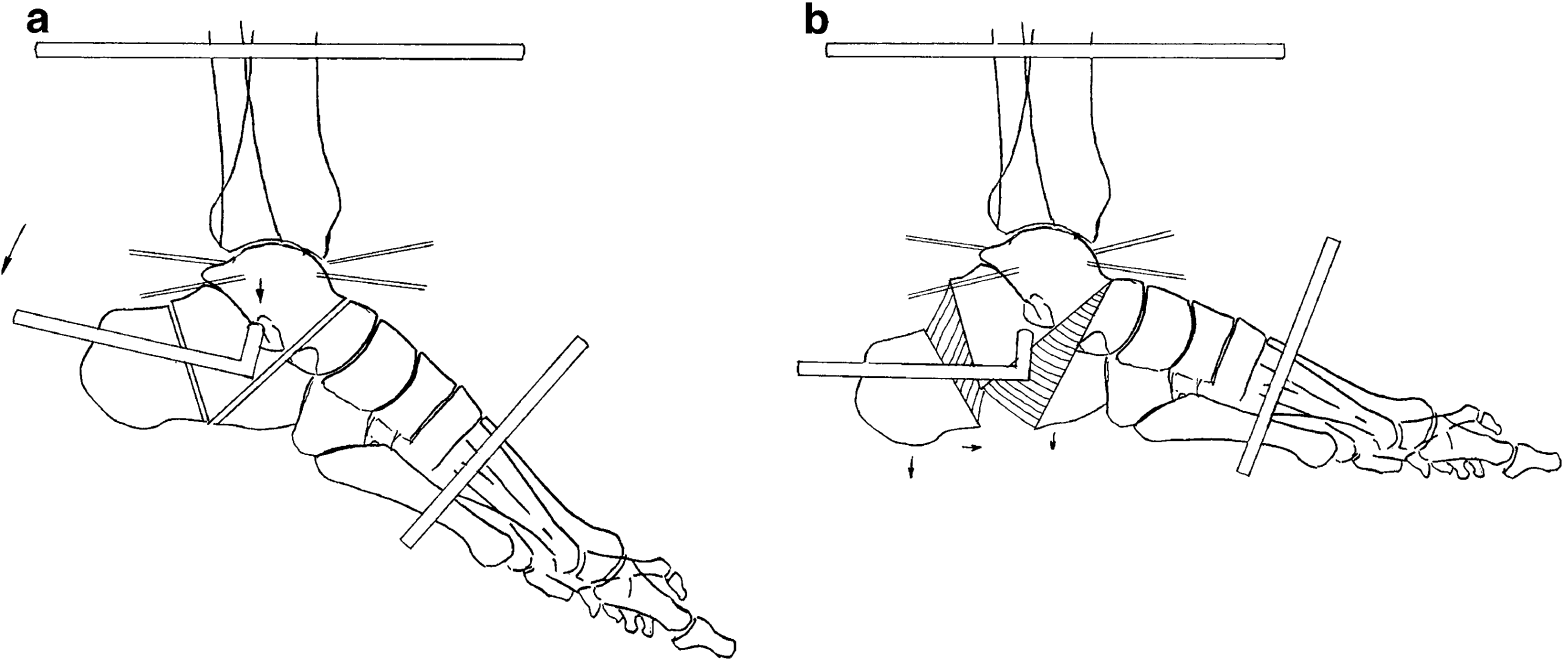
*V-*osteotomy before correction (**a**) and after correction (**b**)

**Fig. 6:**
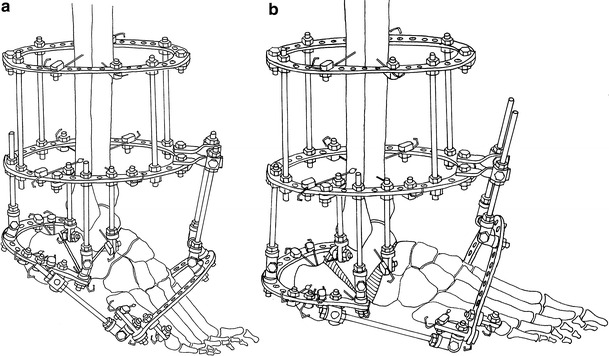
Ilizarov frame with *V-*osteotomy before correction (**a**) and after correction (**b**)

**Fig. 7:**
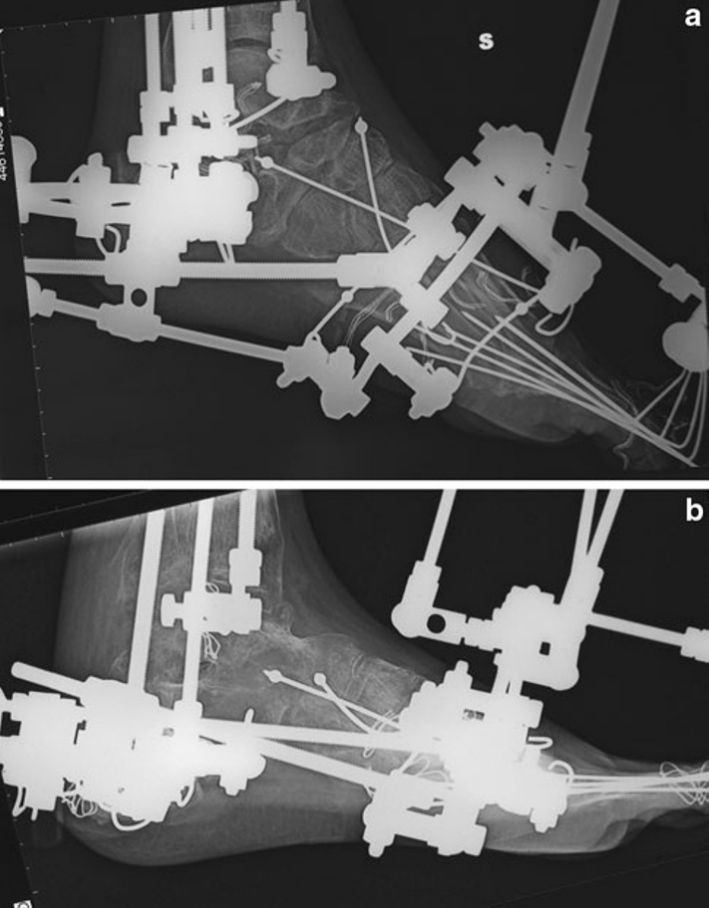
Plain X-ray (lateral view) of the foot and the ankle joint showing *V-*osteotomy and the use olives wires to prevent premature consolidation before distraction (**a**) and after correction (**b**)

**Fig. 8:**
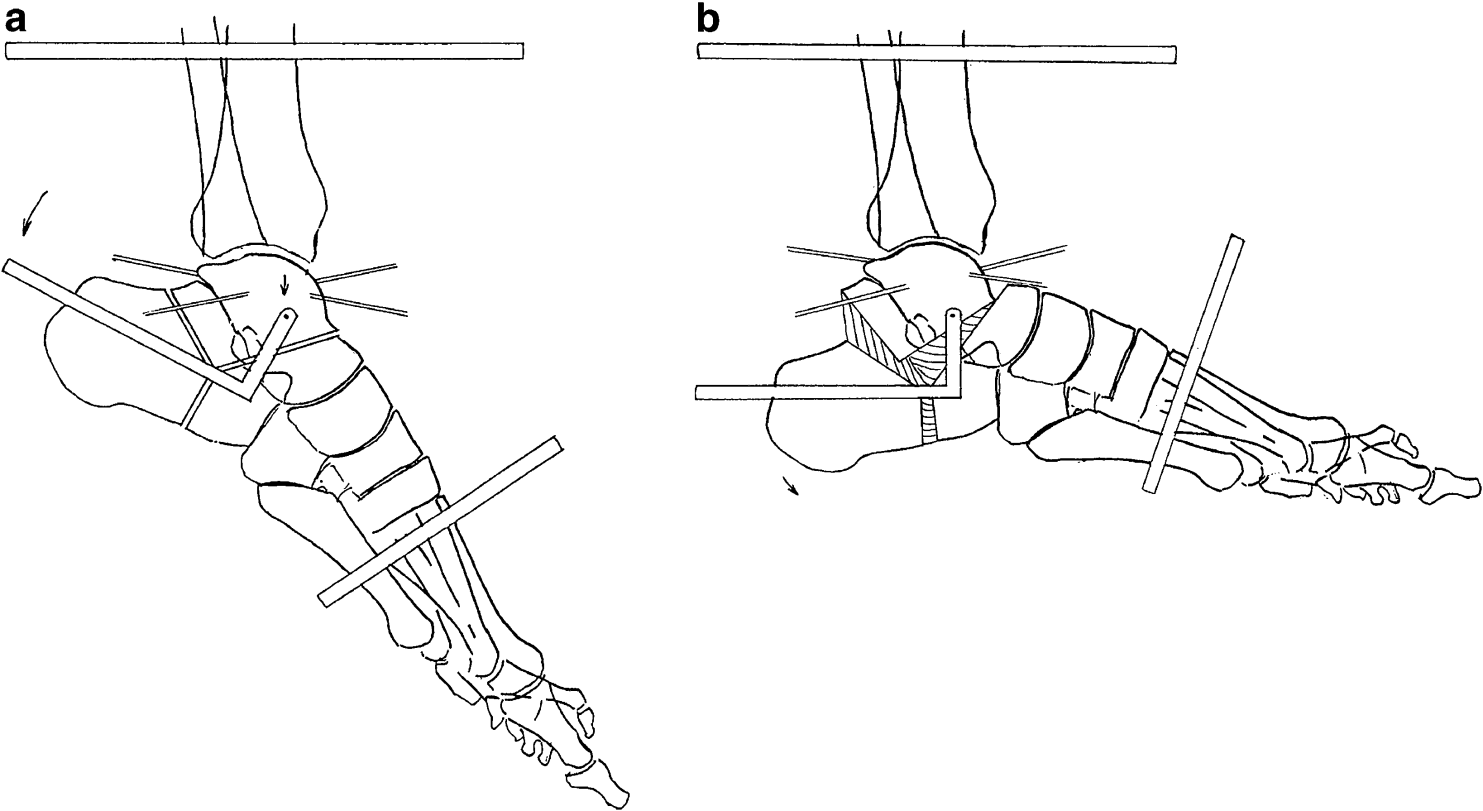
*Y-*Osteotomy before correction (**a**) and after correction (**b**)

Correction is started on the third or fourth day after surgery. The appropriate rods between the tibial assembly and the half-rings should be lengthened at the rate of 1–1.5 mm per day, and the anterior rods between the tibial assembly and the metatarsal half-ring should be shortened at the same rate. The goal is to distract the osteotomies to create the space needed for corrective movement of the bony segments and to prevent premature consolidation.

After 1 week of correction, radiographs are taken in order to observe how the distraction is progressing and to look for subluxations of the tibio-talar joint. In our clinical practice, we take measures to prevent premature consolidation: first, we start distraction on the third day after surgery; secondly, we use cut olive wires inserted into the bone segments adjacent to the osteotomy to enable predictable distraction through the osteotomy instead of distraction of adjacent joints.

#### *Y-*shaped osteotomy

Different to the *V-*shaped osteotomy, the *Y-*shaped osteotomy is exclusively corrective.

##### Operative technique

This technique requires a 2–3 cm lateral and curved submalleolar incision. First, the calcaneus is osteotomized (performing the oblique posterior cut of the Y shape); secondly, a vertical osteotomy of the calcaneus (the vertical cut of the Y shape) and finally a calcaneal-talar neck osteotomy performed. The resulting shape is similar to a three-ray star with the rays equally spaced 120° apart (Figs. 11, 12).

The assembly of the device is the same as the one used for the *V-*shaped osteotomy (Figs. 7, 8). Equinus is corrected by lowering the calcaneus and raising the forefoot with respect to the talar body. The talus is fixed to the tibial assembly with two crossed wires (Fig. 7). The calcaneal half-ring is rotated around the axis of the hinges by the push forces by the posterior rod. The forefoot is simultaneously subjected to proximal traction, in a direction opposite to that of the equinus and the cavus deformities. Supination and equinus are corrected by using different rates of shortening of the two vertical threaded rods between the tibial assembly and the metatarsal half-ring.

### Arthroereisis

Arthroereisis is an operation for limiting excessive motion in a joint with undue mobility from paralysis, usually by means of a bone block. It is a surgical procedure in which the bone adjacent to the joint is modified to limit the motion of that joint mechanically, e.g. at the ankle joint, foot drop is corrected by preventing plantar flexion beyond the plantigrade position with a posterior bone block or, for the treatment of paralytic calcaneus (talipes calcaneus), by limiting the ankle dorsiflexion (anterior arthroereisis). Anterior arthroereisis is indicated for the treatment of a paralytic calcaneus deformity where there is loss of power of the calf muscles and, as a result of stretching of the paralysed muscles, there is an increased range of passive ankle dorsiflexion. The calcaneus posture becomes more and more exaggerated until the heel attains its characteristic pistol shape. Anterior arthroereisis by an anterior bone block may be created by means of the *Y-*shaped osteotomy. Once the osteotomy has been performed and the external fixator assembled, the foot remains fixed in a position of maximum dorsiflexion; in such a position, contact is present between the anterior edge of the tibial epiphysis and the talar neck. After corrective distraction of the anterior branch of the *Y-*shaped osteotomy, this contact remains unmodified but the forefoot position distal to the osteotomy is altered with respect to the tibial axis. In this way, a bone block is formed at the dorsal extension of the foot (Figs. 9, 10, 11, 12, 13, 14, 15, 16). This is not a common procedure but in cases with a stable subtalar and Chopart joint or after triple arthrodesis, it helps to create a stable plantigrade foot. This procedure may be used as an alternative to ankle arthrodesis which, for this type of deformity, is a more drastic procedure.

**Fig. 9:**
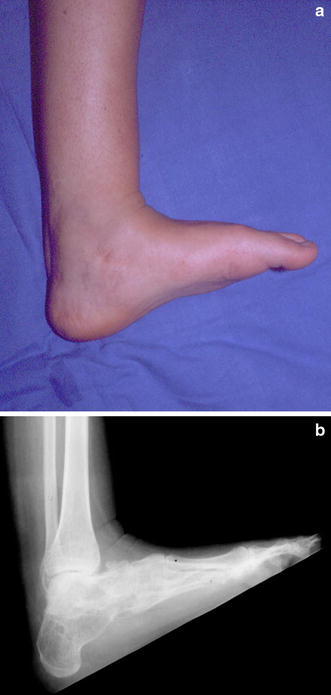
25-year-old female (patient n° 20). Preoperative photograph (**a**) and plain X-ray (**b**) of the poliomyelitic foot with vertical calcaneus deformity (maximum dorsal flexion)

**Fig. 10:**
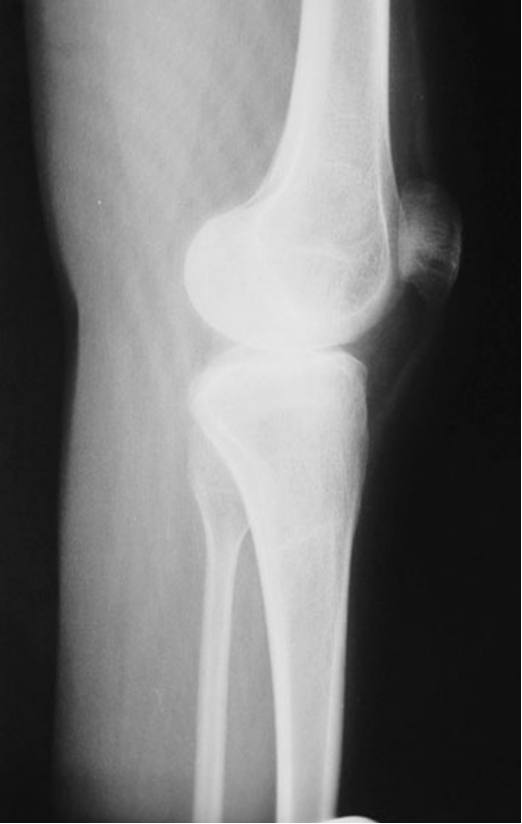
Preoperative plain X-ray showing minor flexion deformity of the knee (lateral view)

**Fig. 11:**
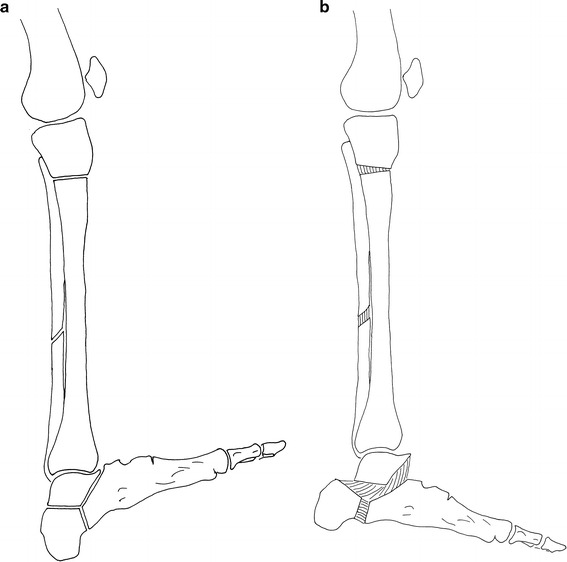
Artrorisi of the foot with proximal osteotomy of the tibia and distal osteotomy of the fibula before correction (**a**) and after correction (**b**)

**Fig. 12:**
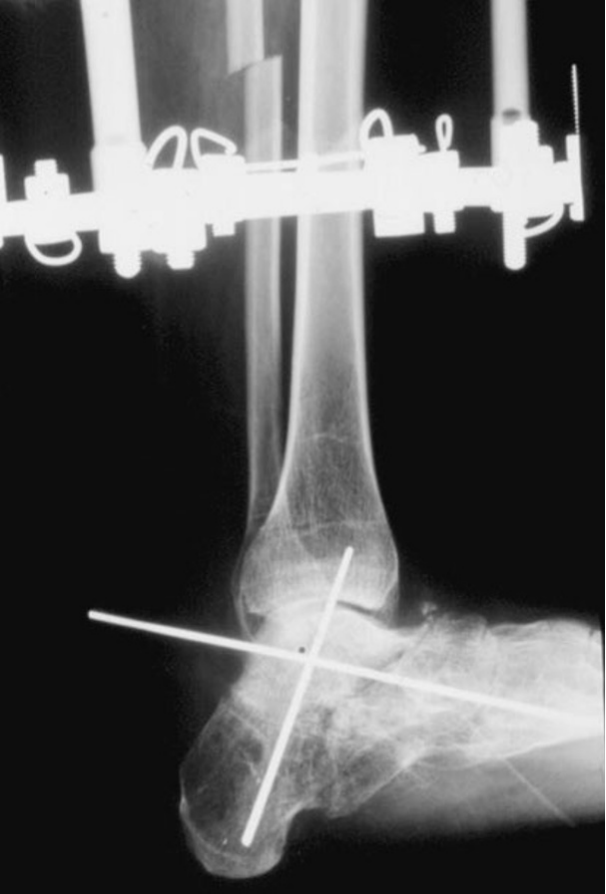
Intraoperative plain X-ray (lateral view) showing *Y-*osteotomy artrorisi with fixation of talus by K-wires

**Fig. 13:**
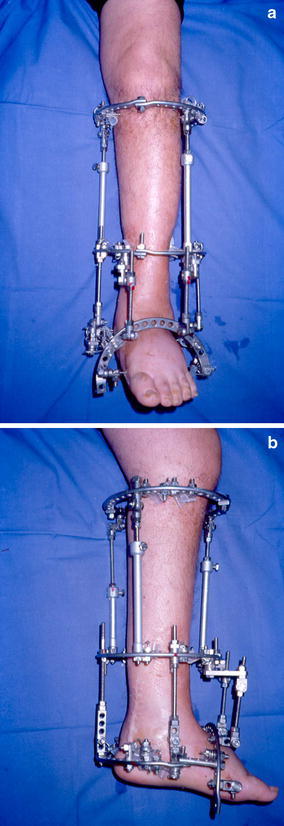
Photograph of the frame during correction: AP view (**a**) and lateral view (**b**)

**Fig. 14:**
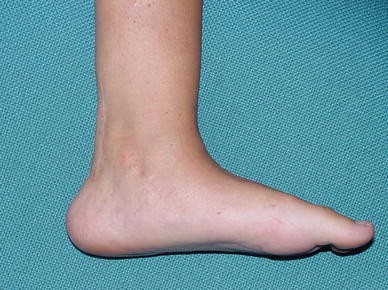
Post-operative photograph of the foot after frame removal

**Fig. 15:**
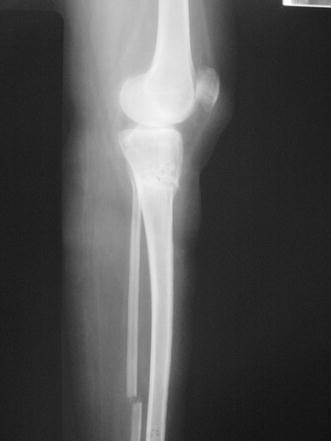
Post-operative plain X-ray showing correction of procurvatum deformity of the knee (lateral view)

**Fig. 16:**
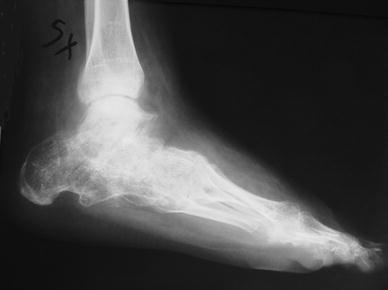
Post-operative plain X-ray of the foot after frame removal (lateral view)

#### Post-operative management

The post-operative management is divided into five different stages:

- Early post-operative phase;
- Distraction phase;
- Consolidation phase;
- Post-frame removal phase;
- Rehabilitation phase.

During the early post-operative phase, the wire, pin and corticotomy sites tend to bleed and this can be reduced by large compression dressing pads. All connection bolts in the frame must be tight, the ends of the wires cut and bent into smooth curls so as not to snag clothing; the Schanz screws are cut as short as possible with the cut ends covered by tape. Pain relief is very important in the early post-operative period as a patient with pain is not able to be cope with physiotherapy and mobilization; to this end, we prefer to use adequate doses of narcotics instead of non-steroidal anti-inflammatory drugs (NSAID) as NSAIDs can affect the osteotomy healing process. Correction of the deformity starts on the third post-operative day while leg lengthening, if this is part of treatment, on the fifth (the day of surgery is being considered as day 0). The process of loosening and tightening threaded rods should be performed four times a day (0.25 mm per time). Patients can return home on the third post-operative day in cases of bloodless technique (fixator application without osteotomy or open procedure) but will need 1 or 2 days longer if an osteotomy or arthrodesis is performed.

During the distraction phase, the patient should be reviewed at least every 2 weeks. During each visit, a detailed clinical examination and X-ray studies are performed. Distance moved along the threaded rods can compared to the position registered during the previous visit. The amount of active and passive ROM of the joints above and below the fixator is recorded. If there is undue redness and pain around pin sites and an associated increase in or a purulent discharge, the patient should start a course of antibiotics. The attending doctor monitors the stability of the fixator and its components. We use X-ray to visualize the distraction gap increasing as desired and changing as progressive correction of the deformity is carried out. Physiotherapy is important during this stage; a variety of exercises are used in order to maintain and improve muscle strength, including open kinetic chain exercises such as quadriceps, hamstring and straight leg exercises. Prevention of anxiety is important; reassurance and encouragement of patients to re-engage in social activities must be kept in consideration.

The consolidation phase can last approximately 45–60 days. Weight bearing with the external fixator is important during the entire period. Customized soles should be used, frequently checked and adjusted during corrective period. The fixator has to be progressively neutralized to ensure that the weight-bearing stresses are transferred to the newly formed bone.

While it is possible to remove the fixator in the outpatient clinic with use of local anaesthesia for olive wire removal in adults, most cases require a short general anaesthetic. In the post-frame removal phase, a plaster cast moulded with overcorrection (and with weight-bearing permitted) is used for 30–60 days for greater security. The patient is encouraged to increase weight bearing gradually, starting with 10–12 kg and increasing at a rate of 2 kg per day.

Physical therapy, exercises for improving range of motion, bracing and gait training have to be done after cast removal during the rehabilitation phase. In our experience, patients feel discomfort in the foot after correction, especially in severe long-standing deformity, but these symptoms tend to remit in a 4- to 6-month period. After correction of an equino-varus deformity, a shoe with a built-up insole (anteriorly and laterally) should be used in order to maintain a 10°–15° overcorrection for a further 40–60 days.

## Results

Our aim was surgical correction in order to produce a stable, painless, plantigrade (defined as a foot at 90°–100° to the limb) and, when possible, a cosmetically acceptable foot. The aim of surgery in polio is for functional improvements and not just a cosmetically normal limb. The mean follow-up period was 7.17 years (range 6 months to 15 years). Table 2 gives details about the type of surgical procedure performed, the period in the external fixator and in a cast for each patient.

**Table 2:** Results details

#	Surgical treatment	Time in frame (months)	Cast (days)	Follow-up	Result complications
1	IL, modified insoles for 2 months, *V-*ost.	6	45	6	Plantigrade foot No complications
2	TAL, IL, percutaneous flexor tendon cut, plantar fasciotomy, STR	4	45	12	Plantigrade foot Clawing toes (after cast removal)
3	TAL, IL, STR	5	60	15	Plantigrade foot No complications
4	IL, tendon transfer (after correction), tibial len., *V-*ost.	8	60	17	Plantigrade adducted foot
5	Ankle arth., IL	6	30	7	Stable plantigrade foot with no complications
6	TAL, IL, modified insoles for 3 months, STR	4	45	18	Plantigrade foot No complications
7	TAL, IL, modified insoles for 3 months, STR	5	45	24	Plantigrade foot No complications
8	TAL, IL, plantar fasciotomy, *V-*ost.	9	45	24	Plantigrade foot Pain after frame removal for 6 months
9	Calcaneal ost., cuboid ost., navicular-cuneiform arth., tibia and fibula ost.	5	0	180	Plantigrade foot No complications
10	STR, tibia and fibula ost., triple arth.	5.5	33	180	Stable foot Toes deformities
11	Proximal and distal tibia ost.	4.8	40	180	Plantigrade foot No complications
12	Distal femur ost., proximal tibia ost., triple arth.	6	35	180	Mild E foot Painful ankle joint
13	Distal femur ost., proximal tibia ost., triple arth.	5.8	31	168	Plantigrade foot No complications
14	Subtalar joint arth., tibia and fibula ost.	4.7	32	168	Plantigrade foot Infection
15	Calcaneal ost., Chopart joint arth., tibia and fibula ost.	4.6	35	168	Plantigrade foot No complications
16	Closed correction of foot deformity, femur ost., tibia and fibula ost.	5.3	0	144	Plantigrade foot
17	Ankle arth., tibia and fibula ost.	6	33	120	Plantigrade foot No complications
18	Tibia and fibula ost., *Y-*ost.	6.3	31	108	Plantigrade foot No complications
19	Tibia and fibula ost., *Y-*ost.	5.2	30	120	Plantigrade foot No complications
20	Proximal tibia ost., *Y-*ost.	4.4	31	120	Plantigrade foot No complications
21	Closed correction of foot deformity, knee arth., tibia and fibula ost.	6.7	35	12	Plantigrade equinus foot
22	Femur ost., tibia and fibula ost., *V-*ost.	7.2	25	84	Plantigrade foot, pain
23	Panarth.	3.5	35	84	Stable equinus foot
24	Ankle joint ankylosis, midfoot ost., tibia len.	5.5	0	60	Ankylosis, broken wires
25	Closed correction of foot deformity, femur ost., tibia ost.	7.3	31	84	Plantigrade equinus foot Pain for 1 year
26	Tibia and fibula ost., triple arth.	6.1	30	18	Varus foot for 1 year Claudication, pain for 1 year, obesity
27	Proximal tibia len., triple arth.	6.3	33	24	Plantigrade foot, flexed knee, difficult walking

*arth* arthrodesis, *IL* Ilizarov frame, *len* lengthening, *ost* osteotomy, *STR* Soft tissue release, *TAL* Tendon Achilles lengthening

There were 6 patients who underwent correction by a closed method (bloodless technique) with the Ilizarov fixator and without any additional procedures. The open method with *V-*shaped osteotomy and calcaneal osteotomy was used in 5 cases, supramalleolar osteotomy in 2 cases, triple and ankle arthrodesis in 3 cases and a Chopart joint arthrodesis in one. The additional procedures performed included limb lengthening in 13 cases, Achilles tendon lengthening in 8 cases, knee arthrodesis and proximal tibial lengthening in 1 case. At the time of fixator removal, a plantigrade foot was achieved in nearly all patients and the leg-length discrepancy eliminated in all cases where it was present. As compared to the preoperative status, all patients were satisfied with their gait.

The correction period ranged from 2 months to 3.5 months. The mean duration of fixator usage was 4.2 months (range 3–7) for the foot portion of the fixator and about 6.1 months (range 5–10) for the tibial portion. In most cases, treatment time depended on consolidation of the tibial segment rather than the foot osteotomy.

We observed residual varus and equinus deformities of the foot in 2 cases that were treated by repeated fixator applications. There were recurrent deformities in 2 patients who were then managed by a *V-*shaped osteotomy and application of the Ilizarov frame. One patient ended up with a stiff ankle and osteoarthritis and was managed by ankle arthrodesis; another patient had significant pain and was managed by physiotherapy.

Complications including pin track infection were infrequent and accounted for 15% of patients; all these patients were treated with oral antibiotics. One patient had a good correction of the foot deformity by triple arthrodesis but had difficulty walking because of weakness of the quadriceps muscles and a flexion contracture of the knee (a brace was fabricated in order to keep the knee in the extended position). Another patient had a knee contracture after femoral lengthening, tibial lengthening and gradual foot deformity correction through a *V-*osteotomy that persisted for 1 year; this patient declined further treatment. In this study, at the time of Ilizarov removal, a plantigrade foot was achieved in 25 cases, mild residual varus and equinus deformities were present in 2 cases, significant pain after removal of the frame, a stiff ankle and recurrent deformity were in one patient, respectively.

## Discussion

The main goal of surgery for patients with foot deformities from polio is to improve the function [16, 17] and not cosmesis. This must be made clear to the patient as some have unrealistically high expectations of what surgery can achieve. There are many options of treatment for foot deformities from poliomyelitis. Traditionally, management of neurological foot deformities required a combination of soft tissue and bony procedures for the correction of the many components of the deformity [4]. Multiplanar foot deformities in neurological disorders that are associated with severe joint stiffness and soft tissue contractures pose a significant challenge to the use of conventional corrective methods, such as osteotomies, triple arthrodesis or talectomy. Acute corrections from large wedge resections of bone lead to a greater risk of damaging the neurovascular bundle, which is usually surrounded by adherent scar tissue. Additionally, these closing wedge corrections and conventional arthrodeses produce additional shortening of the foot and lead to needing shoes of different sizes [9, 18].

The Ilizarov method has some advantages over conventional methods in that it is able to correct the foot deformities, limb-length discrepancy and joint contractures simultaneously. It addresses all the components of the deformity, leading to a stable foot but with lower complication rates. In the Ilizarov method, the osteotomy and progressive correction maintains or even increases the length of the foot and shoe-fitting problems are avoided [9, 16].

The usual corticotomy site for leg lengthening is at the junction of the proximal and the middle thirds of the tibia. Eralp et al. [21] reported that the Gigli saw technique for tibial lengthening gave a lower healing index (HI) in 16 patients with post-poliomyelitic deformities compared with percutaneous multiple drill hole osteotomy. Kristiansen and Steen [22] suggested a bifocal osteotomy for tibial lengthening of more than 6 cm in order to reduce HI and the period of external fixation. The technique of percutaneous osteotomy with an osteotome, as performed in this series, preserves the periosteum and permits an earlier start to distraction (5 days after surgery) at the rate of 1 mm per day in the beginning and 0.75 mm daily subsequently. In this report, we found the healing index was 1.7 for those cases that underwent leg lengthening, a result comparable with other authors’ studies. Ankle equinus occurs frequently during or after the tibial lengthening, and the incidence will depend on the distraction rate, the amount of lengthening, the patient’s age, the aetiology of tibial shortening and the technique of wire or pin insertion. Previous investigators have reported the incidence of ankle equinus after tibial lengthening to vary from 10% to 50% and dependent on the aetiology of shortening [20, 23, 24]. Eldridge et al. [25] and Lehman et al. [26] recommend prophylactic foot fixation during the lengthening phase with a foot frame in order to avoid ankle equinus and its removal during the consolidation phase for patients with tibial lengthening beyond 10% of the original length or more than 6 cm. Huang [19] reports 11 cases of ankle equinus and 2 cases of knee flexion contracture in 35 lengthenings of poliomyelitic tibiae. He recommends prophylactic tendon Achilles lengthening (TAL) as a concomitant procedure during tibial lengthening. In all cases in this series, we fixed the foot in the fixator, which then enabled for the correction of the deformity by the closed method. Song [27] had 4 procurvatum deformities at the proximal tibia in 14 cases during lengthening. We choose to apply the proximal tibial ring obliquely in order to prevent this complication during lengthening of the tibia as we are able to correct procurvatum at the end of distraction.

Several authors have indicated that the Ilizarov method is an effective alternative means of correcting complex foot deformities, especially in feet that have undergone surgery previously [28]. Paley reports 25 complex foot deformities treated by Ilizarov distraction osteotomies, performing more than one type of osteotomy, such as supramalleolar, *U-*shaped, *V-*shaped, posterior calcaneal, talocalcaneal neck, midfoot and metatarsal osteotomies. Satisfactory results were achieved in 22 feet but were unsatisfactory in 3 feet [29].

Several authors have used the *V-*osteotomy for the correction of foot deformities in polio. Kucukkaya et al. report 4 patients who had had poliomyelitis achieving plantigrade feet without recurrence after a *V*-osteotomy for the correction of pes calcaneocavus and simultaneous lengthening at the distal tibia [31]. El-Mowafi H et al. report 25 feet in 15 patients with a complex deformity treated by the Ilizarov method. These were neglected or relapsed clubfeet (13 patients) and foot deformities after poliomyelitis (2 patients). A plantigrade foot was achieved in 18 cases and gait improved in all patients. There were residual varus deformities in 2 patients [30].

We have used arch wire techniques in the simpler cases and for corrective triple arthrodesis where there was separate fixation of hindfoot and forefoot. We noted that during the tibial lengthening, a knee flexion contracture rather than ankle equinus was more likely to occur because of immobilization of the ankle with the Ilizarov frame for foot deformity correction. Aggressive physiotherapy during treatment and after removal of the external fixator usually resolved the knee flexion contracture. However, residual tightness of the gastrosoleus and Achilles’ tendon complex may produce deformation of the callus during physiotherapy and lead to a procurvatum deformity. Limb lengthening with or without deformity correction in skeletally mature patients with old polio is associated with more complications than other types of lengthening [10].

## Conclusion

We have treated 27 patients affected by foot deformities caused by poliovirus infections in the last 15 years. We corrected foot deformities and leg-length discrepancy using the Ilizarov method to achieve a stable plantigrade and painless foot. Several surgical techniques were used (closed method, open method with osteotomy, arthrodesis, leg or Achilles’ tendon lengthening) and the surgical objective accomplished (a stable plantigrade and painless foot) in nearly all our patients with few complications.
